# Distractor Effect of Auditory Rhythms on Self-Paced Tapping in Chimpanzees and Humans

**DOI:** 10.1371/journal.pone.0130682

**Published:** 2015-07-01

**Authors:** Yuko Hattori, Masaki Tomonaga, Tetsuro Matsuzawa

**Affiliations:** 1 Primate Research Institute, Kyoto University, Inuyama, Aichi, Japan; 2 Wildlife Research Center, Kyoto University, Inuyama, Aichi, Japan; UNLV, UNITED STATES

## Abstract

Humans tend to spontaneously align their movements in response to visual (e.g., swinging pendulum) and auditory rhythms (e.g., hearing music while walking). Particularly in the case of the response to auditory rhythms, neuroscientific research has indicated that motor resources are also recruited while perceiving an auditory rhythm (or regular pulse), suggesting a tight link between the auditory and motor systems in the human brain. However, the evolutionary origin of spontaneous responses to auditory rhythms is unclear. Here, we report that chimpanzees and humans show a similar distractor effect in perceiving isochronous rhythms during rhythmic movement. We used isochronous auditory rhythms as distractor stimuli during self-paced alternate tapping of two keys of an electronic keyboard by humans and chimpanzees. When the tempo was similar to their spontaneous motor tempo, tapping onset was influenced by intermittent entrainment to auditory rhythms. Although this effect itself is not an advanced rhythmic ability such as dancing or singing, our results suggest that, to some extent, the biological foundation for spontaneous responses to auditory rhythms was already deeply rooted in the common ancestor of chimpanzees and humans, 6 million years ago. This also suggests the possibility of a common attentional mechanism, as proposed by the dynamic attending theory, underlying the effect of perceiving external rhythms on motor movement.

## Introduction

Until the past decade, it was thought that the ability to synchronize movements to auditory beats was unique to humans [1, 2]. This view has been challenged by recent reports of several non-human animals spontaneously moving in synchrony with external rhythms [3–6]. Recently, we reported that a female chimpanzee named “Ai” spontaneously aligned tapping the keys of an electronic keyboard to an isochronous auditory sequence [7]. Although her response was limited in that rhythmic entrainment appeared in only one of three tempi (i.e., ISI- 600 ms), notably, she showed rhythmic entrainment without any specific training.

Ai had never been trained to pay attention to auditory rhythms, only to tap two keys alternately. Therefore, it may be more appropriate to compare her response with those reported in a previous study on the distractor effect of auditory rhythms on human tapping [8], than with studies in which human participants were instructed to synchronize their tapping to auditory rhythms. While sensorimotor synchronization or rhythmic entrainment involves intentional activity, periodic movement sometimes unintentionally cause intermittent entrainment (also referred to as relative coordination), especially when the stimulus and motor tempi are similar [9].

For instance, one study showed that people who intend to ignore auditory distractor stimuli during self-paced tapping were, nevertheless, influenced by them when the stimulus tempo was similar to the self-tapping tempo [8]. When the interonset interval (IOI) of the distractor stimuli was very close to the self-tapping tempo (differed only by 5% or 0%), taps were shifted to the distractor sequence and caused intermittent phase locking. Interestingly, the stimulus tempo with which the chimpanzee Ai showed spontaneous entrainment (i.e., ISI- 600 ms) was also the closest one to her spontaneous motor tempo [i.e., inter-tap-interval (ITI) = 578.5 ms]. So, this suggests that during intentional left/right alternation movement, unintentional timing perturbations of Ai’s movement were attracted to the distractor auditory stimulus. Therefore, the cognitive mechanism of spontaneous responses to external rhythms might be shared to some extent between chimpanzees and humans.

Such an effect of spontaneous or preferred tempo on processing external rhythms has also been mentioned in a human developmental study. Drake and colleagues [10] found significant correlation between spontaneous tapping tempo and the synchronization rate with simple sequences and music in 6-, 8-, and 10-year-old children and adults. In order to describe the underlying cognitive mechanism, they referred to the dynamic attending theory [11], which describes how the ability to attend to external events in time may arise from internal “attending rhythms.” These internal “attending rhythms” are capable of entraining to external rhythms and targeting attentional energy at expected points in time [11]. Thus, if attention flows from an endogenous rhythmic oscillator, predictive periodic events with a close tempo should be processed more efficiently and might more strongly influence their own rhythmic movement than any other stimulus tempo. If a similar effect of spontaneous tempo exists in chimpanzees, it is possible that the attentional mechanism postulated by the dynamic attending theory is shared between humans and chimpanzees.

The present study compared the distractor effect of perceiving auditory rhythms on self-paced tapping in chimpanzees and humans. Although such unintentional response is different from the intended synchronization, which most previous tapping studies have focused on, we believe that it might represent a critical point to understand evolutionary origins of the cognitive foundation of the advanced human rhythmic ability [12, 13]. Because passive listening to auditory beats affects human motor responses, including corticospinal excitability [14], similarity or differences in the distractor effects of perceiving auditory rhythms on rhythmic movement would help us understand how a strong coupling between auditory and motor related areas in the brain was acquired during human evolution.

Chimpanzees are the phylogenetically closest living relatives of humans, but are unable to speak: their vocal cords are located higher in their throats and cannot be controlled as well as human vocal cords are [15]. Nonetheless, they show various kinds of activities which could be considered prerequisites for human music, including drumming in the wild [16, 17], chorusing between group members [18], and playfully making sounds [19]. Additionally, because they share many behavioral, cognitive, and socio-ecological characteristics with humans, an empirical study of responses to external rhythms in chimpanzees is important for gaining insights into the kind of response or sensitivity that was present in humans before a complex vocal learning ability was acquired [20].

We first compared the spontaneous tapping response to auditory rhythms in chimpanzees and humans using the same procedure and apparatus as in our previous study [7] (i.e., experiments 1 and 2). The only difference was that no auditory feedback was given when keys were tapped, in order to observe the multimodal effect caused by perceiving an auditory rhythm. We also measured the spontaneous motor tempo to examine the relationship between the internal tempo and the tempo that altered their tapping.

Next, we verbally instructed human participants to synchronize their tapping onset to the auditory stimulus onset (i.e., experiment 3), as described in previous tapping studies, in order to compare spontaneous and intended responses to auditory rhythms. We aimed to reveal how selective attention and intention by verbal instructions alter motor responses to external rhythms in humans.

## Methods

### Ethics statement

The experimental protocol was approved by the Animal Welfare and Animal Care Committee of KUPRI (Kyoto University, Primate Research Institute) and the Animal Research Committee of Kyoto University (approval number: 2014–037). All procedures were in accordance with the Japanese “Act on Welfare and Management of Animals.” The care and use of the chimpanzees adhered to the 2002 version of the Guide for the Care and Use of Laboratory Primates by KUPRI. At the time of testing, the chimpanzees lived in a social group of 13 individuals in an enriched environment with a 700- m2 out door compound and an attached indoor residence that was illuminated during daytime. Each compound was connected to the experimental room by a tunnel [21]. The outdoor compound was equipped with climbing frames, ropes, small streams, and various tree species. Access to the outdoor compound was available to them every other day during the day. Daily meals included a wide variety of fresh fruits and vegetables fed throughout the day, and supplemented with nutritionally balanced biscuits. Water was available *ad libitum*.

The human participants provided written informed consent prior to the experiments and received full debriefing upon completion of the study. The reported human studies were reviewed and approved by the Human Ethics Committee, Primate Research Institute, Kyoto University (H2011- 07).

### Participants

We tested three chimpanzees (“Ai,” a 37-year-old female; “Ayumu,” a 13-year-old male; and “Pal,” a 13-year-old female) and six humans (two men and four women, both groups with a mean age of 27 years). None of the human participants had any professional musical training. The chimpanzees had never been trained to respond to any rhythms but had participated in previous studies, including a tapping experiment [7] consisting of auditory stimuli, conditional discrimination between visual and auditory stimuli [22], and multimodal effects of auditory pitch on visual matching-to-sample tasks [23]. However, no previous study required the chimpanzees to adjust their movements in response to auditory rhythms. The chimpanzees were given fruit (i.e., an apple or raisins) as reward.

### Apparatus

We used the same apparatus as that used in [7], including an electronic keyboard (Casio- LK 208, Casio, Japan) with a function to illuminate the keys upon tapping, written in a specific midi channel. We created midi data to indicate the keys to be tapped with light and a chime sounded when the trial finished after the key tapping sequence was completed. The midi data were created using the music edit software, Cubase 6 (Steinberg, Germany) and transferred to the keyboard. For the experiment, the keyboard was connected via Cubase 6 to a computer, which recorded the timing of the tapping in real time and played the distractor auditory stimuli. A previous study suggested that latency is 3.5 ms on average with 3.2 ms peak jitter [24]. However, the feedback sound was not played by tapping the keyboard. After the experiment, the tapping data was saved into midi files and imported into MATLAB (Mathworks, United States) using the MIDI toolbox [25]. The tapping onset times were analyzed with MATLAB Circstat Toolbox [26] and SPSS (IBM, United States).

### Procedure

**Fig 1:**
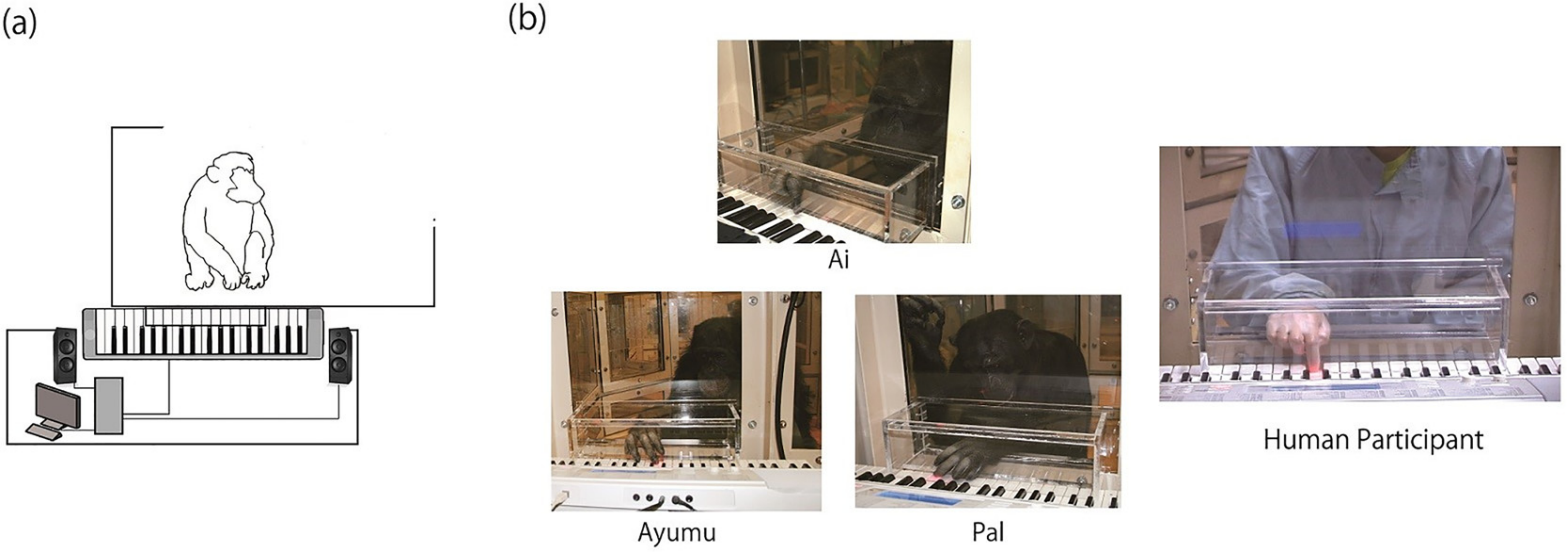
(a) A schematic drawing of the experimental setting and (b) a picture of chimpanzee Ai, Ayumu, and Pal, and a human participant during an experiment.

**Fig 2:**
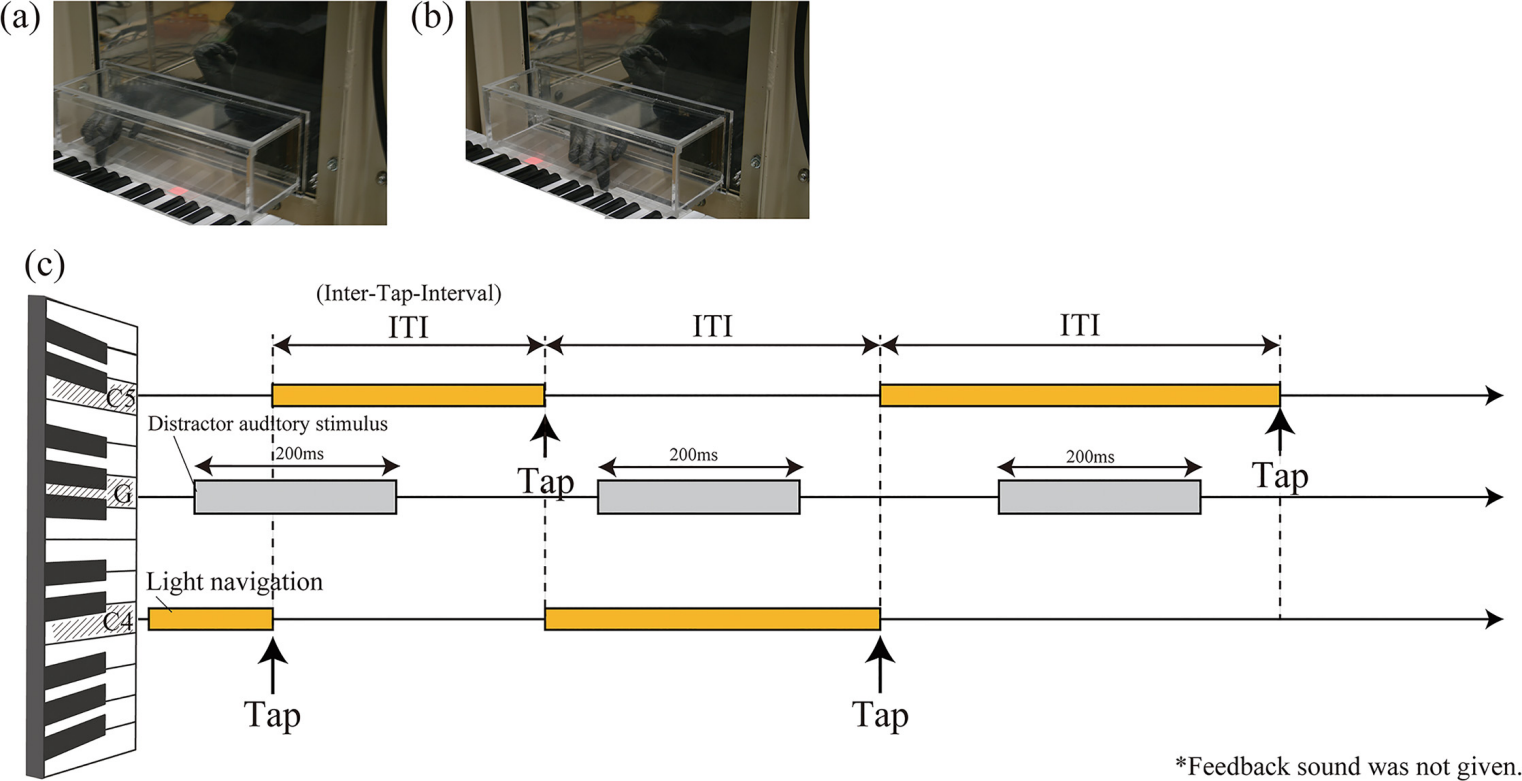
(a) Ai tapped “C4”and (b) “C5.” Note that feedback sound was not provided for tapping. (c) Time sequence of a test trial.

We provided an electronic keyboard to three chimpanzees and six humans (Fig 1) and instructed them to tap two keys (i.e., “C4” and “C5,” see Fig 2) alternately 30 times using the light navigation. At the start of a training trial, a key “G” was lit as a start button. When the participants tapped “G,” another key “C4” was lit. If the participants tapped “C4”, the illumination immediately switched to key “C5” (see Fig 2) and when they completed tapping keys “C4” and “C5” alternately 30 times following the light navigation, a chime (auditory signal for trial completion) was played and the chimpanzees were rewarded.

If the chimpanzees tapped different keys from the lit key, it was counted as a mistake and the same key remained illuminated. However, when they tapped both the lit key and another key, it was counted as a hit and the next key was illuminated. Because light navigation switched to the other key soon after the participants tapped the required key, it is unlikely that the participants learned particular rhythms.

We continued this training until the participants could tap the keys 30 times with fewer than three mistakes (i.e., fewer than 10% error overall) in two consecutive trials. In a test phase, one session consisting of one training trial (the same as in training), followed by three test trials (as in training but with distractor auditory stimuli), was conducted for each condition i.e., ISI: 400 ms, 500 ms, 600 ms, ‘no stimulus’, and ‘random’ [auditory stimulus was played with ISIs randomly chosen from a set of ISIs (300, 400, 500, 600, 700, and 800 ms) with equal probability] in “G” key (392 Hz). The “no stimulus” condition was always conducted first, followed by the other four conditions in a random order. No condition was run more than twice daily. In total, testing was carried out over 6 days, thus, we collected data for 6 sessions (consisting of 18 trials and 540 taps) for each condition. Between each condition, we inserted one training trial to ensure 30 times key tapping regardless of the auditory stimulus.

In the first experiment, we examined whether chimpanzees and humans showed a consistent relationship between tapping timing and onset of each auditory stimulus when hearing an isochronous auditory stimulus (i.e., ISI: 400, 500, and 600 ms) during self-paced tapping. We found significant phase locking in one chimpanzee and three humans when an auditory stimulus was close to their spontaneous tapping rate. Because the spontaneous tapping interval of one chimpanzee (Pal) was faster than 400 ms, we replicated this experiment with faster stimuli (i.e., ISI: 320, 340, 360, and 380 ms) in experiment 2. In the third experiment, we verbally instructed human participants to synchronize their tapping to the auditory stimuli in order to compare the spontaneous responses obtained in experiment 1.

In all experiments, onset asynchrony between tapping and the auditory sound was measured in milliseconds and converted onto a circular scale (in degrees:-180°to +180°) with stimulus onsets aligned at 0°. In experiments 1 and 2, in order to statistically analyze whether auditory rhythms had any influence on tapping timing, we adopted the Hodges-Ajne test (omnibus test) [27] because we observed not only unimodal but also multimodal distribution in the datasets. The Hodges-Ajne test does not assume sampling from specific distributions and works well for unimodal, bimodal and multimodal distributions [27]. We further performed Monte Carlo tests where each tapping onset series with no sound stimulus was randomly paired with a stimulus time series that induced phase locking in order to confirm that phase- locking occurred above the chance level. For trial-by-trial analysis in experiments 1 and 2, and analysis in experiment 3, we adopted the Rayleigh test because a unimodal distribution was expected [28]. We also measured the median and absolute deviation of ITI to analyze the spontaneous tapping behaviors in chimpanzees and humans. For multiple comparisons, *P*-values were adjusted using the Bonferroni correction.

## Results

### Experiment 1: Effect of auditory rhythms on self-paced tapping

#### (Participants: three chimpanzees and six humans)

**Fig 3:**
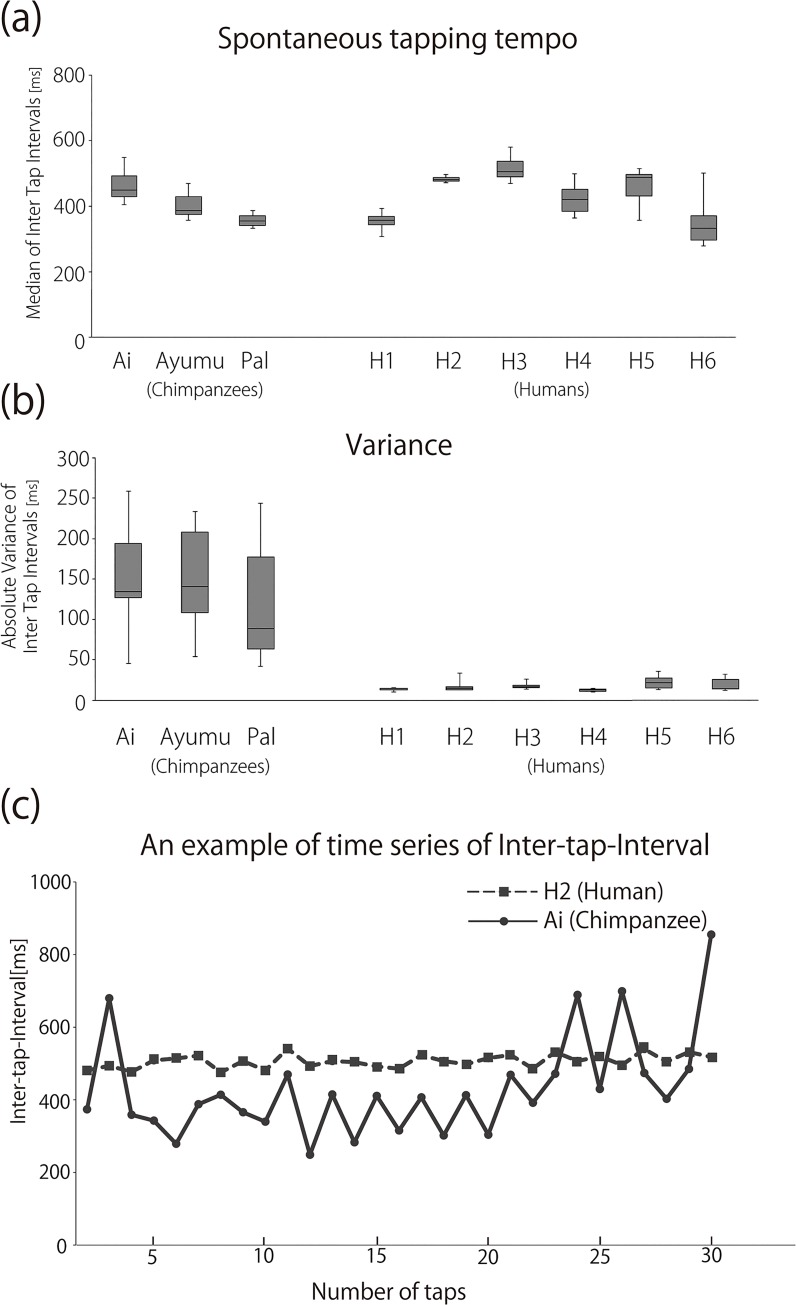
(a) Median inter-tap-intervals and (b) average of absolute variance of chimpanzees and human participants in ‘no stimulus’ condition. The figure shows the average (crossbar), the range from the 25^th^ to 75^th^ percentiles (shaded box), and the minimum and maximum values (thin line). (c) An example of time series of inter-tap-interval by Ai (chimpanzee) and H2 (human). Tapping data was taken from the 1^st^ trial of the ‘no stimulus’ condition.

We played one of the four auditory sequences (i.e., ISI: 400 ms, 500 ms, 600 ms, and random in “G” Key, 392 Hz) or no sound during self-paced tapping by chimpanzees and humans for 18 30-tap trials (total 540 taps) for each condition. In order to determine the spontaneous motor tempo and variance in both species, we analyzed the ITI in the ‘no stimulus’ condition. Overall, both chimpanzees and humans tapped two keys in the range of 400 to 600 ms, with no significant difference between the species (t-test for independent samples, *T* (7) = -0.717, *P* = 0.497) (Fig 3A and Table 1). In contrast, we found a significant difference in variance with ten times larger variances in chimpanzees than in humans (t-test for independent samples, *T* (7) = 17.784, *P* < 0.0001) (Fig 3B and Table 1). Even when outliers (tapping interval that deviates from median ± 2 absolute variances) were excluded, chimpanzees still had six times larger variances than humans. This suggests that chimpanzee tapping fluctuated throughout the trials rather than having long pauses on some occasions within otherwise regular taps (see also Fig 3C).

**Table 1:** Median inter-tap-intervals (ITI) and average of absolute variance of each chimpanzee and human participant while they tapped the keyboard at their self-paced tempo (with No stimuli) in Experiment 1.

	Spontaneous tapping tempo [ms]	Variance [ms]
	(Median ITIs)	(Average of Absolute Variance)
(Chimpanzees)		
Ai	449.5	152.0
Ayumu	386.5	149.5
Pal	355.5	120.9
(Humans)		
H1	358.5	13.4
H2	481.5	17.4
H3	505	18.1
H4	419.5	12.6
H5	487	23.0
H6	333	19.8

Thus, in general, average tapping tempo was similar between chimpanzees and humans, but human tapping was more stable. The same tendency (i.e., similar tapping tempo but larger variance in chimpanzees than humans) was found when we compared the tapping data from chimpanzees in our previous study [7] with those from humans in the current study, although this general difference might be due to past experiences.

**Fig 4:**
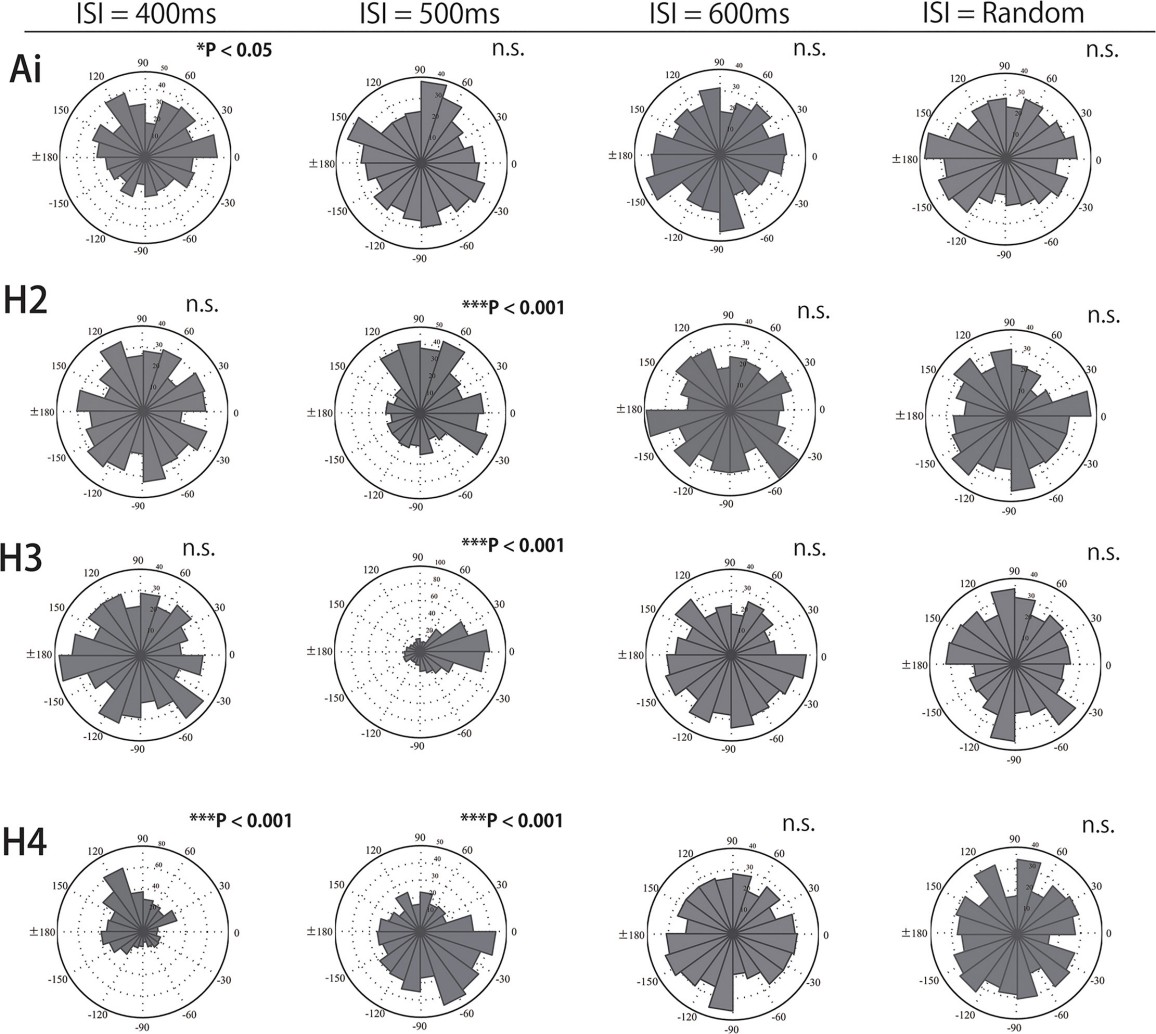
Circular distributions of tapping in Ai, H2, H3, and H4 in ISI 400, 500, and 600 ms, and random conditions.

In order to examine whether the auditory sequences influenced tapping differently in each participant, we conducted Hodges-Ajne tests (omnibus test) for each condition and each participant. Significant skewed distributions were found in one chimpanzee, Ai, and in three human participants (Fig 4). The spontaneous tapping rate of Ai was 449.5 ms, with a consistent relationship with the ISI- 400 ms auditory stimulus that was the closest to her tapping rate (*P* < 0.05, Bonferroni corrected (the correction criterion was 0.000794)). The mean direction was 61.49°, indicating that Ai tapped about 70 ms after the stimulus onset.

We conducted a Monte Carlo test to confirm if this consistency was above the chance level. Because we ran 30 training trials (with no sound stimulus) one between each condition, we randomly selected 18 of these (i.e., 540 taps) without replacement, and re-paired the tapping onset and auditory beat time series of the 400- ms condition. We made 10,000 sets of such data and conducted Hodges-Ajne tests to check for non-uniform distributions in these simulated data. We then computed the *p*-value of the original data (i.e., *P* = 0.000794) as the proportion of the simulated experiments that had the same or higher skewed distribution. The proportion was 0.0048 (i.e., *P* = 0.0048), indicating that the degree of observed phase- locking was matched or exceeded by only 0.48% of the simulated data, and this is highly unlikely to have occurred by chance.

Interestingly, humans showed a similar response to the auditory stimuli. For example, the spontaneous tapping tempi of human participants “H2” and “H3” were 481.5 and 505 ms, respectively, and they responded to the auditory rhythm only in the ISI- 500 ms condition (Hodges-Ajne tests with unspecified direction, *Ps* < 0.0001, Bonferroni corrected (the correction criterion was 0.00000159)). Whereas the mean direction of H3 with ISI- 500 ms was -7.12°, indicating a negative mean asynchrony, the mean direction of H2 with ISI- 500 ms was 45.95°, which is behind the auditory stimulus by approximately 60 ms. Another human participant “H4”, showing a spontaneous tempo of 419.5 ms, tapped in significant skewness with ISI-400 ms and ISI-500 ms beats (Hodges-Ajne tests, *Ps* < 0.0001, Bonferroni corrected (the correction criterion was 0.00000159), see also Fig 4). The mean directions were 128.35° (ISI- 400 ms) and -64.26° (ISI- 500 ms), which also indicates that tapping onset was 142.6 ms after and 89.2 ms before the stimulus onset, for ISI 400 and 500 ms, respectively. In contrast to a previous tapping study[12, 13], the results show various relationships between tapping timing and stimulus onset in human participants. Other participants and conditions showed no specific relationships. The same Monte Carlo tests with 10,000 simulations confirmed that the observed distribution was highly unlikely to have arisen by chance (H2 with ISI- 500 ms: *P* < 0.001, H3 with ISI- 500 ms: *P* < 0.001, H4 with ISI- 400 ms: *P* < 0.001, and H4 with ISI-500 ms: *P* < 0.001).

**Fig 5:**
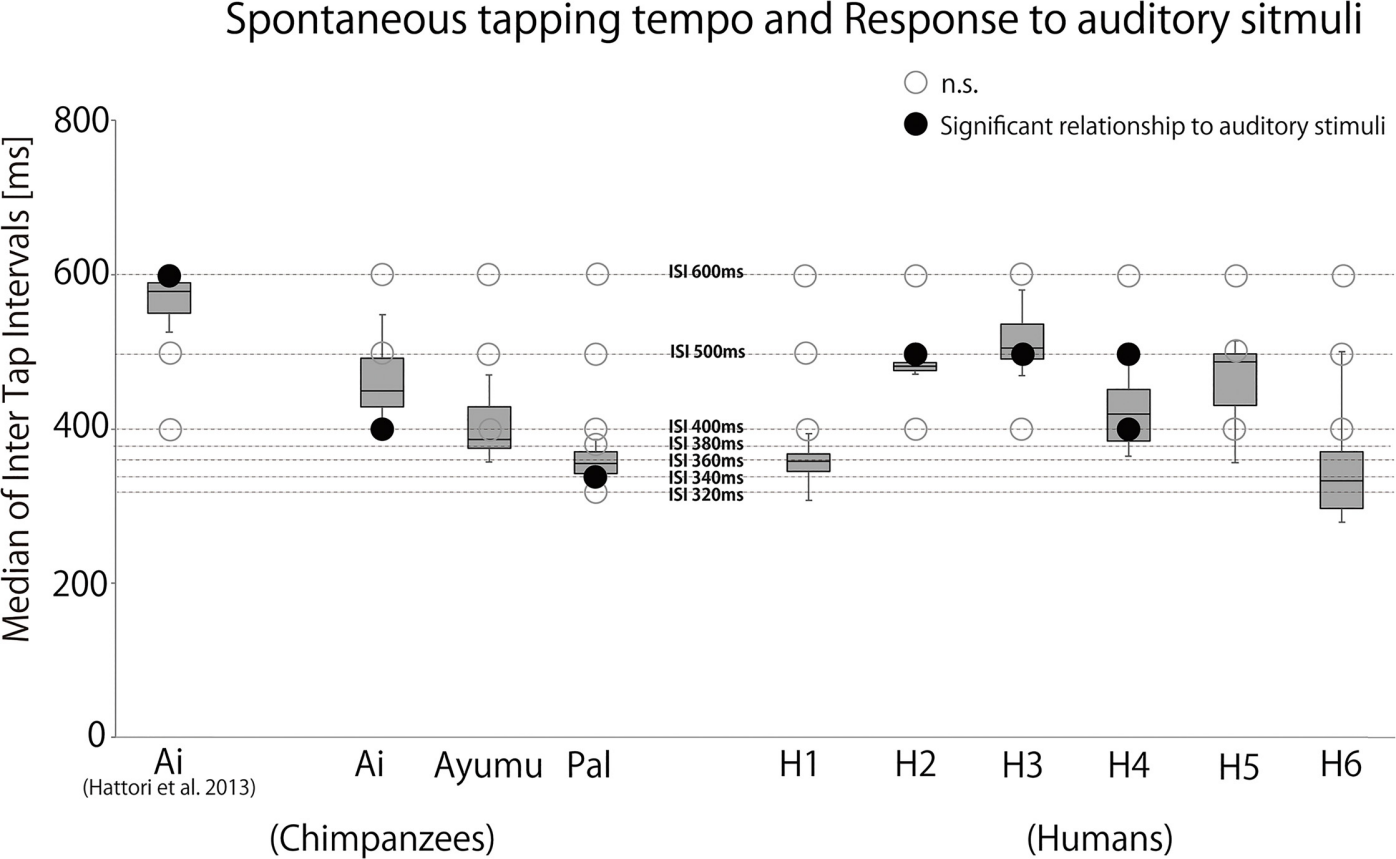
Spontaneous tapping tempo and response to each auditory stimuli. The plotted results also include the tapping results of Ai already published in Hattori, Tomonaga, and Matsuzawa (2013).

The results indicate that chimpanzees and humans can show a similar distractor effect and this effect occurs only when the stimulus tempo is close to the spontaneous motor tempo of the participants (Fig 5). Specifically, the distractor effect occurred only when the spontaneous motor and auditory stimulus tempi differed only by less than 20%. Negative mean asynchrony was absent in some cases. Our results do not agree with previous tapping studies containing instructions to synchronize to auditory rhythms, suggesting that selective attention and intention might have affected the response to external rhythms. Therefore, without selective attention and intention, even humans may not necessarily show accurate synchronization. This suggestion is supported by a recent study showing that activation in motor areas involved in pulse perception is weak when attention was not focused on auditory stimuli [29].

**Fig 6:**
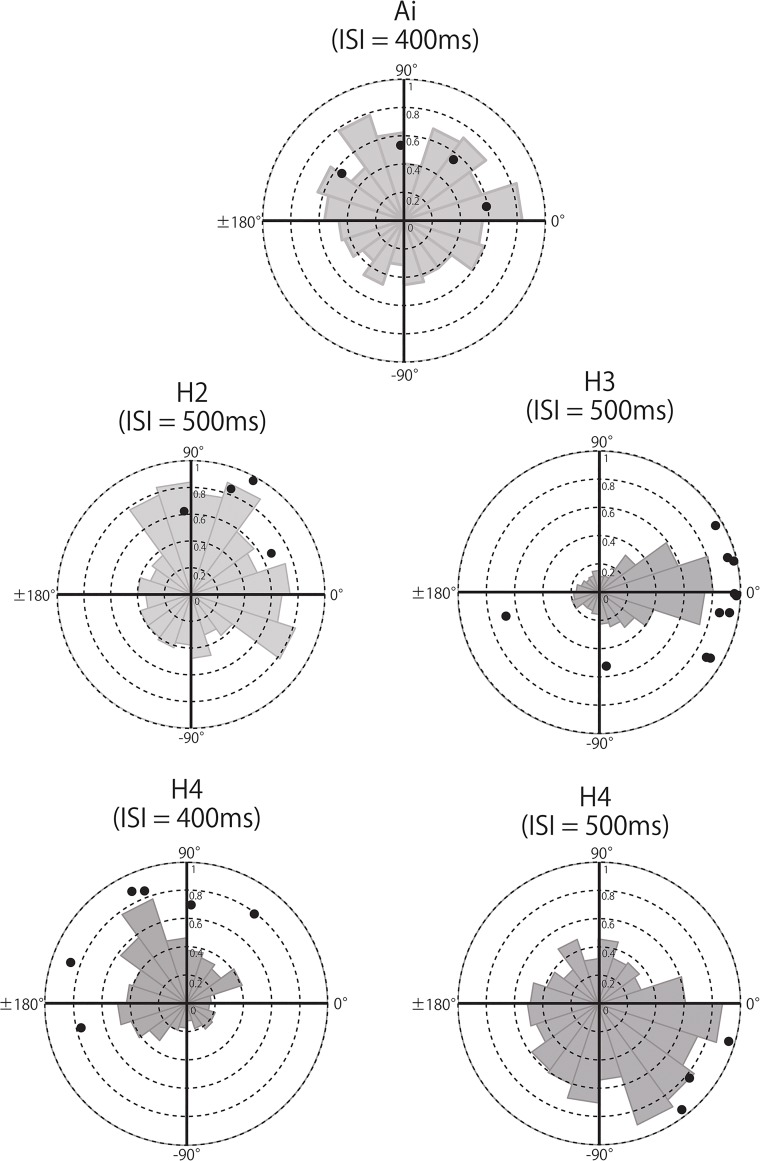
The mean vector lengths of trials that reached significance in Rayleigh tests. We plotted the significant trials from the total 18 trials. We conducted this detailed analysis in those conditions that showed significance in the Hodges-Ajne test (omnibus test). The background is the circular distribution of each datasets. We also compared the dataset in the ‘no stimulus’ condition with the auditory beat sequence to determine whether significant uniformity might occur by chance.

Until now, the spontaneous response to auditory rhythms has not been much investigated in tapping studies. Therefore, we conducted a trial-by-trial analysis using the Rayleigh test in order to investigate whether phase- locking appeared over all trials or intermittently across trials (i.e., 18 trials in total). For the chimpanzee Ai, significant phase-locking was found in 4 of the 18 trials (Rayleigh tests with unspecified direction, *Ps* < 0.05, Bonferroni corrected (the corrected criterion was 0.000278)). The mean directions ranged from 9.49°to 143.31°with a significant consistency overall, and a mean direction of 61.49°related with local intermittent entrainment in 4 trials (Fig 6). We conducted the same analysis for the ‘no stimulus’ condition compared with the ISI- 400 ms sequence, but no significant effects emerged. Thus, intermittent entrainment is highly unlikely to have occurred by chance during self-paced tapping by Ai.

In humans, the same analysis revealed significant phase- locking in4, 11, 6, and 3 trials for H2 in ISI- 500, H3 in ISI- 500, and H4 in ISI- 400 and ISI- 500 ms conditions, respectively (Rayleigh tests with unspecified direction, *Ps* < 0.05, Bonferroni corrected (the corrected criterion was 0.000278)) (Fig 6). However, similar trends occurred in the ‘no stimulus’ condition compared with the auditory sequences (3, 5, 2, and 1 trial in the ‘no stimulus’ condition for H2 and H3 compared with the ISI- 500 ms sequence, and H4 compared with ISI- 400 and 500 ms sequences) (Rayleigh tests with unspecified direction, *Ps* < 0.05, Bonferroni corrected (the corrected criterion was 0.000278)). However, the average score of vector length in each condition was significantly longer when participants tapped with auditory rhythms than with ‘no stimulus’ (*T* (3) = 3.64, *P* = 0.036). Therefore, the phase- locking which appeared in humans cannot be explained either by simply tapping stably at a self-paced tempo.

Overall, our results show that the distractor effect appears in both species when the stimulus tempo is close to their self-tapping tempo and differ only by less than 20%. This is consistent with a previous study [8] showing that when human participants are instructed to ignore auditory distractor beats, intermittent phase- locking still occurs with a tempo close to the preferred tempo (5% or 0%). Therefore, characteristics of an unintentional response to auditory rhythms are shared to some extent between chimpanzees and humans.

Phase locking was not found in the other chimpanzees, such as Pal, possibly because the stimulus tempo was too distant from her spontaneous motor tempo (i.e., 355.5 ms). If a stimulus tempo was closer to her own rate, it might have influenced her tapping. We tested this in the next experiment.

### Experiment 2: Effect of auditory rhythms close to the spontaneous motor tempo

#### (Participant: one chimpanzee–“Pal”)

**Fig 7:**
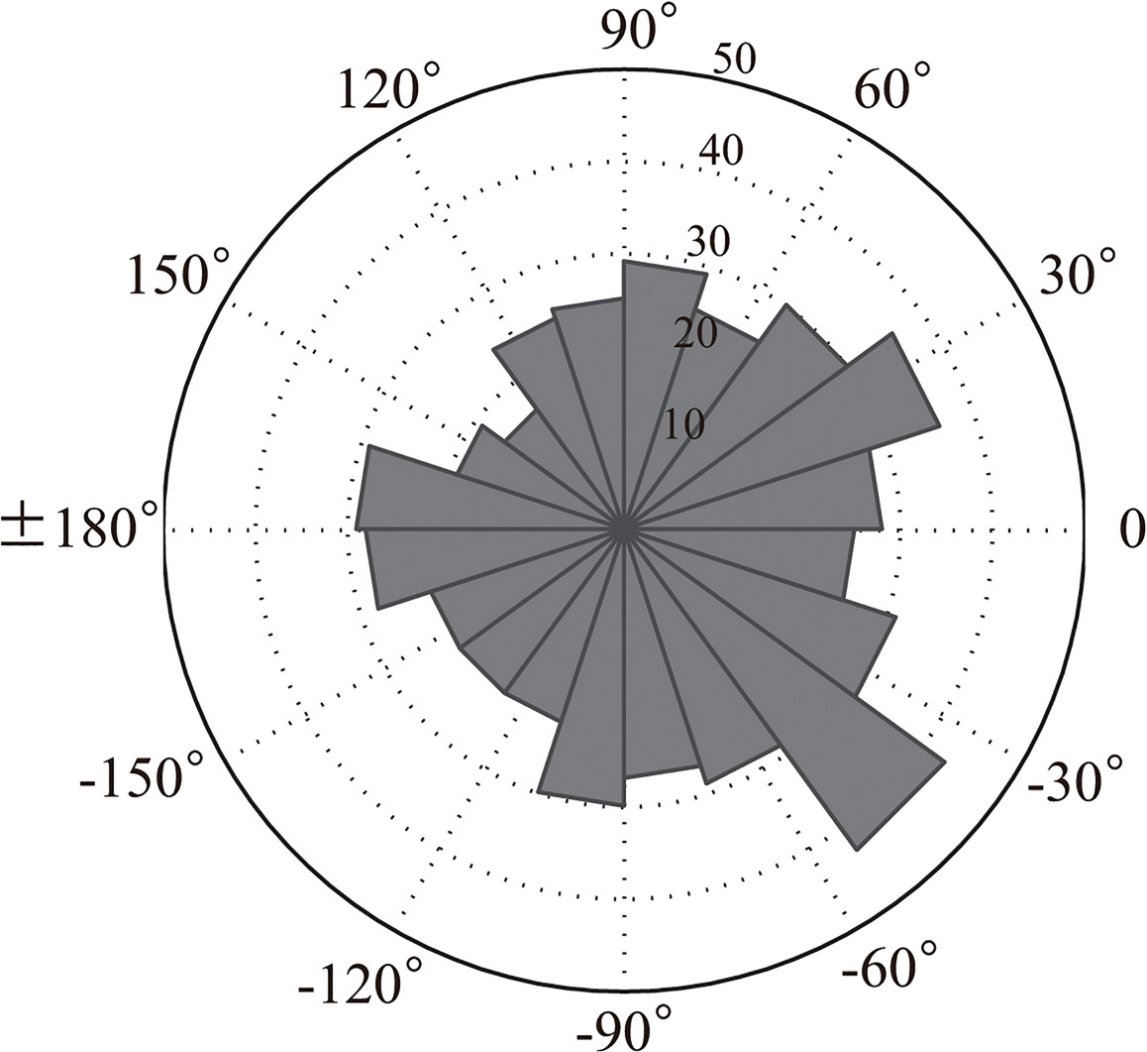
Circular distributions of tapping by Pal in ISI- 340ms condition, for which Hodges-Ajne test showed a significant non-uniformity in the distribution.

We replicated experiment 1 but used auditory tempos closer to the spontaneous motor tempo (i.e., 355.5 ms) of the chimpanzee Pal (i.e., ISI: 380, 360, 340, and 320 ms).This difference apart, the procedure and analysis were the same as in Experiment 1. As expected, we found a significantly consistent relationship between tapping and auditory onsets when the ISI beat was 340 ms (Hodges-Ajne test, *P* < 0.05, Bonferroni corrected (the correction criterion was 0.0056), see also S1 Video) (Figs 5 and 7). The same Monte Carlo test as in experiment 1 with 10,000 simulations confirmed that this is highly unlikely to happen by chance (*P* = 0.0037).

The mean vector direction was 112.71°, indicating that the onset of tapping was behind the stimulus onset by approximately 110 ms. No significant effect was found for any other conditions including ‘random’ and ‘no stimulus’ conditions compared with the auditory sequences (i.e., ISI: 380, 360, 340, and 320 ms). Thus, phase- locking also appeared in Pal when the stimulus tempo was close to her self-paced tapping tempo. The absence of any effect in the ‘no stimulus’ condition when compared with the auditory sequence also rejects the null hypothesis that the consistent relationship found in ISI-340 ms condition happened by chance.

Similar to experiment 1, we conducted trial-by-trial analysis using the Rayleigh test for ISI- 340 ms and the ‘no stimulus’ condition with ISI- 340 ms sequence. Four trials in the ISI- 340 ms condition and 2in the ‘no stimulus’ condition showed a significant relationship between tapping and auditory onsets (*Ps* < 0.05, Bonferroni corrected (the corrected criterion was 0.00389)). The average score of the mean vector length of trials was significantly longer when they tapped with the auditory rhythm than with ‘no stimulus’ (*T* (4) = 3.549, *P* = 0.024). Therefore, the phase- locking which appeared in Pal is also highly unlikely to have happened by simply tapping stably at a self-paced tempo.

### Experiment 3: Instructed synchronized tapping to auditory rhythm

#### (Participants: six humans)

In order to compare spontaneous responses to auditory rhythms in experiment 1 and intended responses to synchronize it in humans, we conducted another experiment with a verbal instruction to synchronize the tapping onset to the auditory stimulus (i.e., ISI: 400, 500, and 600 ms). The same human participants and procedures as in experiment 1 were used.

**Fig 8:**
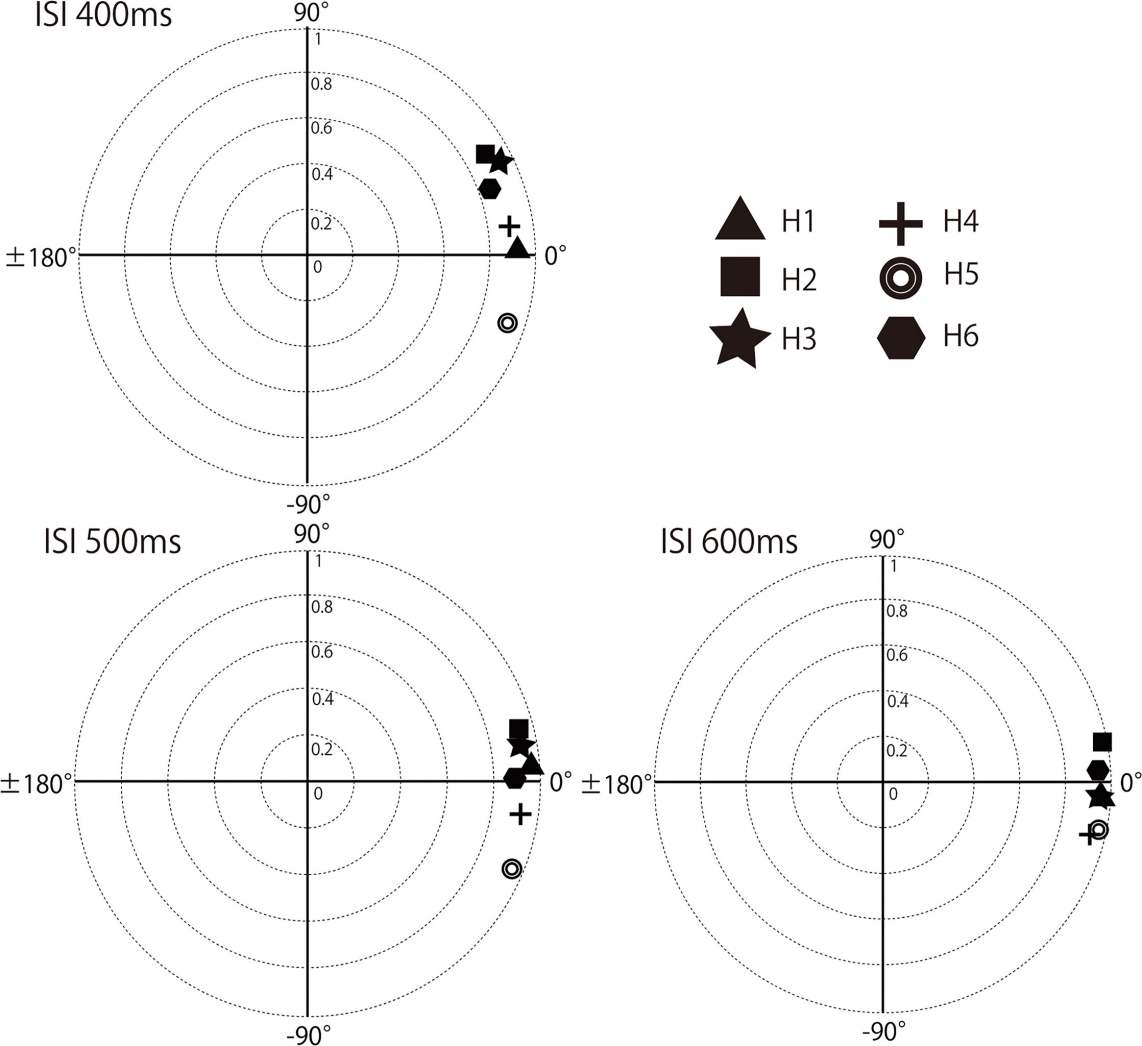
The mean vector lengths of the datasets in all three conditions by all human participants in experiment 3. A dataset consisted of 540 taps. A significant relationship with auditory rhythms was found in the datasets of all human participants in all conditions.

Rayleigh tests with unspecified direction revealed that all participants flexibly synchronized their tapping to all of the stimuli (Rayleigh tests with unspecified direction, *Ps* < 0.0001, Bonferroni corrected (the correction criterion was 0.0028)). The mean directions were around 0°, showing a negative mean asynchrony in some participants (i.e., 1, 2, and 5 participants in ISI- 400, ISI- 500, and ISI- 600ms conditions; see also Fig 8 and Table 2). The lack of negative mean asynchrony in the other participants might be because the movement required in this study (i.e., tapping two keys alternatively) is different from that of previous tapping studies (i.e., tapping one key repeatedly)[9, 13]. Including an oscillating movement as well as a discrete tapping movement might have led to a larger variance in this experiment [13]. This result differs from that of experiment 1 (Fig 8), and suggests that verbal instruction affects the attention level, and leads to flexible and accurate auditory-motor tapping. We did not test chimpanzees in this respect because the purpose of our study was to directly compare spontaneous responses. Whether chimpanzees can learn to intentionally synchronize their movement to external rhythms should be investigated in future studies.

**Table 2:** Mean vector direction and mean resultant length of each human participant in experiments 1 and 3.

	Mean direction (°)			Mean resultant length		
ISI	400 ms	500 ms	600 ms	400 ms	500 ms	600 ms
Experiment 1						
H2	-	45.95	-	-	0.19	-
H3	-	-7.12	-	-	0.4	-
H4	128.35	-64.23	-	0.29	0.2	-
Experiment 3						
H1	1.22	3.67	-3.32	0.92	0.97	0.97
H2	29.65	13.93	10.06	0.89	0.94	0.97
H3	25.95	9.84	-3.56	0.94	0.93	0.96
H4	7.84	-8.32	-14.11	0.89	0.92	0.94
H5	-18.72	-23.1	-12.48	0.93	0.96	0.97
H6	19.8	0.75	3.09	0.87	0.89	0.95

## Discussion

The present study is the first direct comparison of the distractor effect of perceiving auditory rhythms in chimpanzees and humans. Although the effect was weak, tapping onset was spontaneously influenced in both species by an auditory stimulus when it was close to the spontaneous tapping tempo. Such an effect might be due to a connection between an auditory processing area and a related motor area in the brain. Presently, we do not have any evidence that chimpanzees flexibly align their movements to auditory rhythms like in human dancing or singing; thus, such a connection might be much stronger in humans than chimpanzees. However, the connection itself might have arisen gradually [30], and could be due to factors other than complex vocal learning [5, 6, 31], because chimpanzees have limited vocal learning abilities.

What kind of adaptive function would connection between auditory and motor brain areas serve in chimpanzees? We suggest that auditory-motor coupling could bestow advantages in the context of the complex social environment of chimpanzees. Several researchers have suggested that behavioral synchrony may function as a bonding mechanism [32, 33], possibly facilitating the representation of self and others as a whole, and enhancing empathy and/or affiliation [34]. Chimpanzees also show advanced social intelligence through cooperation [35, 36], prosocial behaviors [37], and empathetic perspective taking [38]. Therefore, rhythmic entrainment may have played a role in their advanced sociality and increased group size [32].

Although further studies in a wider range of primate species are necessary, this “social-cohesion hypothesis” seems consistent in primates. For example, the marmoset, a New-World monkey with a highly cooperative breeding strategy, also exhibits vocal turn-taking including coupled oscillator dynamics [39], which is similar to the dynamics proposed for human conversational turn-taking [40]. Marmosets and other platyrrhine primates diverged from the lineage that led to humans and apes approximately 35–40 million years ago, so this characteristic may have evolved in parallel with human rhythmic entrainment. Nonetheless, it supports the hypothesis that cooperative communication has evolved advanced sensitivity and responses to external rhythms.

In contrast, recent studies suggest that macaque monkeys, phylogenetically closer to humans but with less highly cooperative behaviors than marmosets, have difficulty aligning their behavior with external rhythms [41]. Although sensitivity to simple [42] but not complex rhythm [43] exists, whether perceived auditory rhythms can be transferred to the motor realm is unclear. Further studies are necessary, but differences between macaques and humans may indicate a relationship between sociality and rhythmic entrainment.

We also propose that the effect of spontaneous tapping tempo might be explained by the dynamic attending theory and a common attentional mechanism underlying the effect of motor movement of perceiving external rhythms in chimpanzees and humans. When humans were instructed to pay attention to an auditory rhythms, all participants flexibly synchronized their tapping to the stimulus. Rhythmic synchronized activity by chimpanzees in natural conditions has never been reported; therefore, advanced synchronized movement with a wide range of external rhythms might have emerged during human evolution, although further empirical work is necessary to reach a conclusion.

In general, communication allows individuals to coordinate their behavior, and it facilitates cooperation. If individuals are motivated to have stronger relationships, increasingly sophisticated ways of exchanging information might evolve. Because music and dance are advanced tools that connect human individuals, strong motivation for bonding could be one of the factors that facilitated the evolution of rhythmic entrainment. Although our study did not directly focus on such advanced rhythmic ability, the results suggest that a foundation for perceiving and responding to external rhythms is shared between chimpanzees and humans, both highly social primate species. Further studies of temporal characteristics in primate communication, as well as direct comparisons of rhythmic sensitivity between humans and non-human animals would help to uncover how we came to dance and sing together during our evolution.

## Supporting Information

S1 Video Tapping in synchronized with ISI 340 ms stimuli by chimpanzee “Pal”. (MOV)
